# Evaluation of the Performance of a Point-of-Care Test for Chlamydia and Gonorrhea

**DOI:** 10.1001/jamanetworkopen.2020.4819

**Published:** 2020-05-14

**Authors:** Barbara Van Der Pol, Stephanie N. Taylor, Leandro Mena, Joel Lebed, Candice Joy McNeil, LaShonda Crane, Aaron Ermel, Adam Sukhija-Cohen, Charlotte A. Gaydos

**Affiliations:** 1 Department of Medicine, University of Alabama at Birmingham School of Medicine, Birmingham; 2 Department of Medicine, Louisiana State University Health Sciences Center, New Orleans; 3 Department of Medicine, University of Mississippi Medical Center, Jackson; 4 Planned Parenthood of Southern Pennsylvania, Philadelphia; 5 Department of Medicine, Wake Forest University Health Sciences, Winston-Salem, North Carolina; 6 Planned Parenthood Gulf Coast, Houston, Texas; 7 Department of Medicine, Indiana University School of Medicine, Indianapolis; 8 AIDS Healthcare Foundation, Los Angeles, California; 9 Department of Medicine, Johns Hopkins University School of Medicine, Baltimore, Maryland

## Abstract

**Importance:**

Rates of chlamydial and gonococcal infection continue to increase in the United States, as do the associated costs of untreated infections. Improved diagnostic technologies that support testing and treating in 1 clinical visit are critical to advancing efforts to control the rates of chlamydial and gonococcal infection.

**Objective:**

To evaluate the clinical performance of a point-of-care (POC) molecular diagnostic assay for the detection of chlamydia and gonorrhea.

**Design, Setting, and Participants:**

A noninterventional, cross-sectional clinical study was conducted from September 18, 2018, through March 13, 2019, at sexually transmitted infection (STI), HIV, family planning, and obstetrics and gynecology clinics where STI screening is routine, using a convenience sample and comparing commercially available assays with a new 30-minute POC assay. Patients included were those eligible for STI screening or diagnostic testing who had not taken antibiotics effective against chlamydia or gonorrhea within the previous 28 days. Four vaginal swab samples were collected from women and a first-catch urine sample was obtained from men.

**Main Outcomes and Measures:**

A composite infection status was used to classify participants as infected if 2 or more comparator results were positive, as not infected if 2 or more comparator samples were negative, and as unevaluable if 1 result was invalid and the other 2 results did not agree with each other.

**Results:**

Swab samples from 1523 women (median age, 27 years [interquartile range, 17-37 years]), 817 (53.6%) of whom presented with symptoms, and 922 men (median age, 29 years [interquartile range, 17-41 years]), 308 (33.4%) of whom were symptomatic, were tested. For chlamydia, sensitivity of the new POC assay was 96.1% (95% CI, 91.2%-98.3%) for women and 92.5% (95% CI, 86.4%-96.0%) for men. For gonorrhea, sensitivity estimates were 100.0% (95% CI, 92.1%-100.0%) for women and 97.3% (95% CI, 90.7%-99.3%) for men. For chlamydia, specificity of the new POC assay was 99.1% (95% CI, 98.4%-99.5%) for women and 99.3% (95% CI, 98.4%-99.7%) for men. For gonorrhea, specificity estimates were 99.9% (95% CI, 99.5%-100%) for women and 100% (95% CI, 95.5%-100%) for men. Non–laboratory-trained personnel performed 94.8% of all tests (2318 of 2445) during the study.

**Conclusions and Relevance:**

This study suggests that self-obtained vaginal swab samples were associated with performance equivalent to laboratory-based molecular diagnostics, which can support use of this POC assay in many settings. The availability of an easy-to-use, rapid (30-minute) molecular test for accurate detection of chlamydia and gonorrhea has the power to facilitate testing and treatment in a single patient visit for these STIs.

## Key Points

**Question:**

How does a new point-of-care assay for detection of chlamydia and gonorrhea compare with commercially available laboratory-based molecular diagnostics?

**Findings:**

This cross-sectional study including 1523 women and 922 men found that a new molecular point-of-care assay was associated with excellent performance compared with laboratory-based molecular diagnostics for vaginal swab samples and has been cleared for use by the US Food and Drug Administration. Male urine samples were associated with good performance in this assay and are undergoing continuing evaluation.

**Meaning:**

Highly sensitive, rapid chlamydia and gonorrhea testing at the point of care is now a possibility that can support same-day testing and treatment strategies.

## Teaser

This cross-sectional study evaluates the clinical performance of a point-of-care molecular diagnostic assay for the detection of chlamydia and gonorrhea.

## Introduction

Over the last several years, there have been sustained increases in rates of infection with *Chlamydia trachomatis* and *Neisseria gonorrhoeae* (CT/NG) in the United States as reported by the Centers for Disease Control and Prevention (CDC).^1^ Since 2013, the rate of chlamydia infection has increased by 25%, while the rate of gonococcal infection has increased by 74% despite the availability of highly sensitive and specific laboratory-based molecular diagnostic tools for detection of these sexually transmitted infections (STIs).^1,2^ This increase is concerning because these STIs can increase HIV transmission^3^ and have the potential to increase rates of pelvic inflammatory disease (PID)^4,5^ and tubal factor infertility^6^ among untreated women. These preventable sequelae of untreated chlamydia and gonorrhea infections are estimated to cost the US health care system billions of dollars per year.^1^

These increases in rates, despite national recommendations^7,8^ for at least annual screening of women younger than 25 years, men who have sex with men, and people taking HIV preexposure prophylaxis, coincide with reductions in funding for STI control programs and specialized STI clinical services.^9^ As a result of the reduced capacity of STI clinics to provide services that include screening, primary care professionals are expected to cover the gap in services. However, according to the Healthcare Effectiveness Data and Information Set, at least one-half of women in the eligible age range who have an encounter with the health care system are not screened per CDC guidelines.^10^

Further exacerbating our inability to control these STIs is the current reliance on either patients returning to receive treatment after laboratory test results are available or patients receiving treatment based on epidemiologic prediction of the best treatment based on clinical signs and patient-reported symptoms. In the first case, there are delays of usually 7 to 10 days between the initial office visit and treatment based on laboratory test results,^11^ which introduces the potential during that interval for infections to be transmitted to sexual partners and provides an opportunity for the development of PID.^12^ To avoid these outcomes, many practices, including STI clinics and emergency departments,^13^ provide epidemiologic treatment, or syndromic management, for chlamydia and gonorrhea. This practice can lead to undertreatment and overtreatment of chlamydia and gonorrhea. Treating for chlamydia or gonorrhea when the organisms may not be present should be discouraged for good antibiotic stewardship.^13^

Strategies that facilitate adherence to guidelines and inform immediate and appropriate treatment are urgently needed to have an effect on the current state of the STI pandemic. A new US Food and Drug Administration (FDA)–cleared, molecular point-of-care (POC) diagnostic technology has been developed that is easy to use, supports rapid (30-minute) access to highly accurate chlamydia and gonorrhea detection, and can be performed by non–laboratory-trained personnel in clinical settings. We report the performance characteristics of this new assay and suggest potential applications designed to improve chlamydia and gonorrhea screening and diagnostic services.

## Methods

The study was a noninterventional prospective cross-sectional comparison study, with all comparator samples collected from each participant, conducted from September 18, 2018, through March 13, 2019. Women and men seeking STI screening or diagnosis at 11 clinics in 9 geographical locations (Baltimore, Maryland; Birmingham, Alabama; Winston-Salem, North Carolina; Houston, Texas; Indianapolis, Indiana; Los Angeles, California; Jackson, Mississippi; New Orleans, Louisiana; and Philadelphia, Pennsylvania) were recruited. All study procedures were reviewed and approved by the institutional review board routinely used by each collection site (University of Alabama at Birmingham School of Medicine, Louisiana State University Health Sciences Center, University of Mississippi Medical Center, Planned Parenthood of Southern Pennsylvania, Wake Forest University Health Sciences, Planned Parenthood Gulf Coast, Indiana University School of Medicine, AIDS Healthcare Foundation, and Johns Hopkins University School of Medicine). These collection sites included STI, HIV, family planning, and obstetrics and gynecology clinics where STI screening and diagnostic testing are routinely performed. Written consent was obtained from all participants prior to any study-related procedures. This report is consistent with the Standards for Reporting of Diagnostic Accuracy (STARD) reporting guideline for dissemination of clinical study results.^14^

People who had taken antibiotics effective against chlamydia or gonorrhea within the past 28 days were excluded. Participants were categorized as symptomatic if they reported abnormal discharge, dysuria, genital itching, pelvic pain, or discomfort during intercourse. Women were asked to provide 4 vaginal swabs: 3 clinician-collected swab samples using collection and transport devices approved for use with the 3 commercially available nucleic acid amplification tests (NAATs) used as comparators (Aptima Combo 2, Hologic; ProbeTec Chlamydia Q^x^/Gonorrhea Q^x^, Becton Dickinson; and, cobas 4800 CT/NG, Roche) and 1 swab sample using the collection device for the binx health *io* CT/NG assay (binx health). Women were randomized such that one-half collected their own swab samples for the experimental assay prior to any other swab samples, while the remainder had the experimental assay swab samples collected by a clinician as the last vaginal sample taken. The comparator assay swab sample collection order was randomized.

Men provided a first-catch urine sample that was aliquoted into 4 transport devices: 1 for the new POC assay and the other 3 for the comparator NAATs. Per agreement with the FDA, the total sample size was based on calculating the number of participants needed to identify at least 100 chlamydial infections among both men and women and at least 45 gonococcal infections among both men and women, which was site prevalence dependent. The FDA guidance includes performance targets of 95% sensitivity with a lower-bound 95% CI of 90%, and clearance is based on these targets in combination with other performance features. Additional details of the study design, including the protocol and statistical analysis plan, can be found at ClinicalTrials.gov (NCT03071510).

Samples were stored at 2 to 25 °C if tested within 24 hours or at 2 to 8 °C if the *io* CT/NG assay was performed within 7 days after collection. Testing staff, who received approximately 5 minutes of training on instrument operation, scanned the barcode identifier of the sample into the *io* instrument, mixed the *io* collection tube contents, transferred 500 μL of the tube contents into the *io* cartridge using a fixed-volume disposable pipet provided with the test kit, placed the cartridge into the instrument, and pressed “start.” Total hands-on time was less than 1 minute. Qualitative (positive, negative, or invalid) results were available in approximately 30 minutes.

Comparator assays, chosen because they are the 3 most commonly used chlamydia and gonorrhea diagnostic assays in the United States, were performed in a central laboratory in compliance with the manufacturers’ instructions for use. Technologists performing any given assay (binx or comparator assays) were blinded to the results of the other assays.

Participants were classified as infected based on a composite infection status (CIS) that required at least 2 of the 3 comparator results to be positive. Women with 2 of 3 negative results were classified as uninfected. One woman had 1 invalid result and 2 discordant results (1 positive and 1 negative) generated by the 3 comparator tests; thus, her infection status could not be classified. This participant was excluded from analysis.

Point estimates for the sensitivity and specificity of the new POC assay were calculated and 95% CIs were determined using the Score method.^15^ Comparison of the sensitivity and specificity of the new POC assay with that of each of the comparator assays was performed using Cochran *Q* test, which controls for paired samples, as an omnibus test to assess differences between assays, with a Wilcoxon signed rank test to assess pairwise differences where appropriate. To determine whether study site, clinician or patient collection of vaginal swab samples, or the presence or absence of symptoms was associated with the performance of the new assay, logistic regression and Fisher exact tests were performed. All *P* values were from 2-sided tests, and results were deemed statistically significant at *P* < .05.

## Results

We enrolled 2791 participants, of whom 5 withdrew and 341 were excluded. The 2445 eligible participants comprised 1523 women (median age, 27 years [interquartile range, 17-37 years]) and 922 men (median age, 29 years [interquartile range, 17-41 years]). Most women (817 [53.6%]) and a substantial proportion of men (308 [33.4%]) presented to the clinic with symptoms of an STI. Exclusions occurred because of ineligibility (n = 28), improper collection (n = 16), improper sample storage (n = 274), improper testing (n = 22), or an invalid result from the new POC assay (n = 1) (Figure). A total of 2446 tests were performed, but samples from only 2445 patients generated a usable CIS; thus, only 2445 tests on the new platform were evaluated. Of the 2445 tests performed on the new device, 2318 (94.8%) were tested by non–laboratory-trained clinical staff, such as receptionists, medical assistants, and nurses, who had received approximately 5 minutes of training on performing the assay. There were no adverse events reported during the study.

**Figure.**
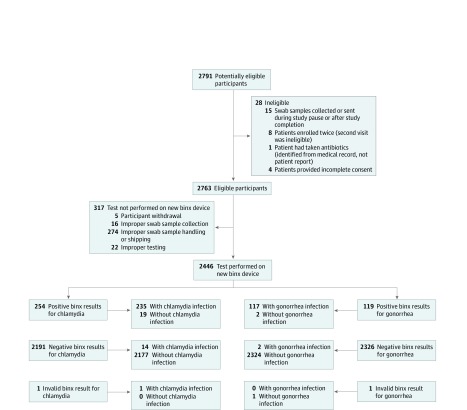
Recruitment and Evaluation Flowchart The flowchart shows the reasons for loss to analysis and the final data disposition numbers.

Samples were obtained from 1523 women, of whom 129 (8.5%) were infected with chlamydia and 45 (3.0%) were infected with gonorrhea (Table 1). For women, all 3 comparator assays were CIS positive in 92.2% (119 of 129) of chlamydial infections and 86.7% (39 of 45) of gonococcal infections, with the remainder of samples generating CIS positives from 2 of the 3 comparator assays (eTable 1 in the Supplement). Positivity rates by symptoms, clinical type, and region of recruitment are shown in Table 1. The new POC assay correctly identified 124 of 129 (96.1%; 95% CI, 91.2%-98.3%) chlamydial infections and 45 of 45 (100%; 95% CI, 92.1%-100%) gonococcal infections (Table 2). Specificity estimates for vaginal swab samples were 1381 of 1394 (99.1%; 95% CI, 98.4%-99.5%) for chlamydia and 1476 of 1478 (99.9%; 95% CI, 99.5%-100%) for gonorrhea. Within the study population, the positive predictive values were 90.5% for chlamydia and 95.7% for gonorrhea, and the negative predictive values were 99.6% for chlamydia and 100% for gonorrhea. The assay performance was not significantly associated with the presence or absence of symptoms nor by patient vs clinician sample collection. However, the chlamydia sensitivity point estimate for self-collected swab samples among symptomatic women was lower (19 of 21 [90.5%]) than that of clinician-obtained swab samples from symptomatic women (40 of 41 [97.6%]). This factor was not a statistically significant difference (Fisher exact test, *P* = .26) and was likely associated with the small sample size in these cells. The sensitivity of the new POC assay using vaginal swab samples was not significantly different from calculated performance estimates for the comparator NAATs (eTable 2 in the Supplement).

**Table 1.** Patient Population and Positivity Rates Based on Composite Infection Status

Characteristic	No. of patients with positive results/No. of patients tested (%)			
	Women		Men	
	Chlamydia	Gonorrhea	Chlamydia	Gonorrhea
Total evaluated	129/1523 (8.5)	45/1523 (3.0)	120/922 (13.0)	74/922 (8.0)
Symptom status				
Symptomatic	62/817 (7.6)	29/817 (3.5)	60/308 (19.5)	62/308 (20.1)
Asymptomatic	67/706 (9.5)	16/706 (2.3)	60/614 (9.8)	12/614 (2.0)
Clinic type				
STI	33/388 (8.5)	19/388 (4.9)	86/569 (15.1)	58/569 (10.2)
HIV	No women^a^	No women^a^	12/193 (6.2)	9/193 (4.7)
Family planning or OB/GYN	96/1135 (8.5)	26/1135 (2.3)	22/160 (13.8)	7/160 (4.4)
Region				
South	121/1314 (9.2)	34/1314 (2.6)	71/485 (14.6)	33/485 (6.8)
East	3/135 (2.2)	3/135 (2.2)	15/167 (9.0)	16/167 (9.6)
Midwest	5/74 (6.8)	8/74 10.8)	24/89 (27.0)	16/89 (18.0)
West	No women^a^	No women^a^	10/181 (5.5)	9/181 (5.0)

Abbreviations: OB/GYN, obstetrics and gynecology; STI, sexually transmitted infection.

^a^ Sites only enrolled men.

**Table 2.** Performance of *io* CT/NG Assay Using Vaginal Swab Samples for Detection of Chlamydia and Gonorrhea Compared With the Composite Infection Status

Characteristic	No.	Chlamydia				Gonorrhea			
		Sensitivity		Specificity		Sensitivity		Specificity	
		No./total No. (%)	95% CI	No./total No. (%)	95% CI	No./total No. (%)	95% CI	No./total No. (%)	95% CI
Self-obtained asymptomatic	349	33/33 (100)	89.6-100	314/316 (99.4)	97.7-99.8	11/11 (100)	74.1-100	337/338 (99.7)	98.3-99.9
Self-obtained symptomatic	387	19/21 (90.5)	71.1-97.3	362/366 (98.9)	97.2-99.6	10/10 (100)	72.2-100	377/377 (100)	99.0-100
Clinician-collected asymptomatic	357	32/34 (94.1)	80.9-98.4	320/323 (99.1)	97.3-99.7	5/5 (100)	56.6-100	352/352 (100)	98.9-100
Clinician-collected symptomatic	430	40/41 (97.6)	87.4-99.6	385/389 (99.0)	97.4-99.6	19/19 (100)	83.2-100	410/411 (99.8)	98.6-100
Total	1523	124/129 (96.1)	91.2-98.3	1381/1394 (99.1)	98.4-99.5	45/45 (100)	92.1-100	1476/1478 (99.9)	99.5-100

Abbreviation: CT/NG, *Chlamydia trachomatis* and *Neisseria gonorrhoeae*.

Among the 922 eligible men, 120 (13.0%) chlamydia infections and 74 (8.0%) gonococcal infections were identified (Table 1). All 3 comparator assays were CIS positive for chlamydia from 110 of 120 male urine samples (91.7%), and the remainder of CIS-positive samples had 2 positive results from the 3 comparator assays. For gonococcal infections, 69 of 74 positive samples (93.2%) generated positive results by all 3 assays, and the remainder were positive in 2 of the 3 assays (eTable 3 in the Supplement). The sensitivity estimates for the experimental assay were 111 of 120 (92.5%; 95% CI, 86.4%-96.0%) for chlamydia and 72 of 74 (97.3%; 95% CI, 90.7%-99.3%) for gonorrhea (Table 3). Specificity estimates were 796 of 802 (99.3%; 95% CI, 98.4%-99.7%) for chlamydia and 848 of 848 (100%; 95% CI, 95.5%-100%) for gonorrhea. Within this population, positive predictive value estimates were 94.9% for chlamydia and 100% for gonorrhea, and negative predictive value estimates were 98.9% for chlamydia and 99.8% for gonorrhea. Male urine performance was not associated with symptom status. Male urine sensitivity using the new POC assay was lower than laboratory-based tests, which were all 99% or higher (eTable 4 in the Supplement).

**Table 3.** Performance of *io* CT/NG Assay Using Male Urine for Detection of Chlamydia and Gonorrhea Compared With the Composite Infection Status

Symptom status	No.	Chlamydia				Gonorrhea			
		Sensitivity		Specificity		Sensitivity		Specificity	
		No./total No. (%)	95% CI	No./total No. (%)	95% CI	No./total No. (%)	95% CI	No./total No. (%)	95% CI
Asymptomatic	614	56/60 (93.3)	84.1-97.4	549/554 (99.1)	97.9-99.6	11/12 (91.7)	64.6-98.5	602/602 (100)	99.4-100
Symptomatic	308	55/60 (91.7)	81.9-96.4	247/248 (99.6)	97.8-99.9	61/62 (98.4)	91.4-99.7	246/246 (100)	98.5-100
Total	922	111/120 (92.5)	86.4-96.0	796/802 (99.3)	98.4-99.7	72/74 (97.3)	90.7-99.3	848/848 (100)	99.5-100

Abbreviation: CT/NG, *Chlamydia trachomatis* and *Neisseria gonorrhoeae*.

## Discussion

*C trachomatis* and *N gonorrhoeae* remain the 2 most frequently reported notifiable diseases in the United States despite decades of STI control efforts. We need new tools and strategies to make progress toward reducing these STIs that result in substantial personal and economic burden. One such important tool will be POC tests that support the provision of single-visit testing and treatment clinical services. Previously commercialized POC assays relied on antigen detection; while they had advantages compared with culture (eg, not requiring viable organisms and supporting testing and treatment) at the time they were developed and approved by the FDA, in the molecular era we now know that the sensitivity of these assays is likely as low as 40% to 60%. Furthermore, antigen-based assays were for chlamydia only, as there has never been an FDA-approved antigen assay for detection of gonorrhea. Therefore, public health agencies, such as the World Health Organization, developed syndromic management guidelines to allow testing and treatment without the use of any diagnostic assays while simultaneously calling for improved POC tests. Syndromic management is known to be highly inaccurate^16^ and, by definition, does not support sexual health care, such as screening, among asymptomatic people.

Mathematical modeling suggests that molecular POC testing can overcome current programmatic hurdles, such as loss to follow-up and lag time between care-seeking behavior and eventual treatment. In this model, adoption of a POC test with 90% sensitivity was associated with a substantial reduction in the number of infections and in the number of cases of PID.^17,18^ Conceptually, the ability to provide same-day results is an improvement, but it does not always result in testing and treatment because patients may not remain in the clinic until their results are available.^19^ One solution to providing faster results is to have patients collect their own samples prior to any interaction with a clinician (eg, immediately after registering on clinic arrival). This scenario has been evaluated using the 90-minute GeneXpert CT/NG assay (Cepheid), with limited success.^19^ Although patients found self-sampling prior to clinician interactions acceptable, only 21% remained in the clinic for the required time until their results were available. In contrast, a previous study reported that using the new POC assay evaluated in this study, with a sample-first strategy in a local student health clinic, added a mean of only 11 minutes to the visit, and 84% of patients were willing to wait up to 20 minutes.^20^ With clinical workflow reorganization and a 30-minute test, we are now well positioned to realize a true paradigm shift for diagnosing symptomatic patients and screening women who are asymptomatic and require screening according to professional guidelines.

The performance characteristics of the POC assay (equivalent to laboratory-based molecular NAAT assays for vaginal swab samples), ease of use, small footprint (27.5 × 38.4 cm), and rapid (30-minute) time to results will efficiently support a single-visit testing and treatment paradigm. This strategy has been demonstrated to have several advantages. First, testing and treatment can lead to increased clinic capacity by reducing the need for follow-up visits to receive results and treatment, which also offers a cost-savings benefit.^21^ This process can also support improved antimicrobial stewardship. Studies have found that syndromic or epidemiologic treatment of people with symptoms of an STI is highly inaccurate, with overtreatment for some infections as well as missed treatment.^13,16^ However, treatment based on accurate diagnostic results, particularly molecular diagnostics, can be targeted to cover only identified infections, thus reducing unnecessary treatments.^22^ Testing and treatment can also reduce sequelae of untreated infections. Symptoms of PID developed between having a sample collected and returning for treatment for 13% of adolescents in one study.^5^ These episodes could have potentially been avoided using a testing and treatment approach. Finally, revised clinical patient flow and adoption of a highly accurate POC assay, such as the one evaluated in this study, will pave the way for adoption of future assays that can determine the resistance or susceptibility profile of any infections detected. This change will lead to targeted, appropriate treatment, particularly for gonococcal infections, that can enhance antimicrobial stewardship efforts.

### Limitations

This study has some limitations. The sensitivity of male urine for detection of chlamydia was lower than the target performance metric of 95%, with a lower 95% CI of 90%. Although there is room for improvement in this aspect of assay performance, the utility of treating infected patients during the initial clinic visit is well recognized. In a study published more than 20 years ago, a test with sensitivity as low as 65% had demonstrable utility if the rate of return for treatment was low.^23^ Based on this analysis, a POC test with sensitivity of 92.5% will have both clinical and public health utility. The other limitation of this study is that it was not designed in such a way that the assay was eligible for Clinical Laboratory Improvement Amendments waiver review. However, more than 94% of testing was performed by nonlaboratory staff in clinical settings, suggesting that Clinical Laboratory Improvement Amendments waiver status is attainable.

## Conclusions

The availability of a highly accurate POC assay for detection of chlamydia and gonorrhea that is easy to perform may encourage adoption of testing in clinical settings that have not previously adopted a routine screening process. One can now envision young women going into their physician’s office for routine reproductive health care visits and being asked to provide a self-obtained vaginal swab sample for their routine sexual health screening. Destigmatizing STI testing by creating a positive, normative, and convenient process for this type of screening has the potential to move us toward the goal of 100% adherence to the Healthcare Effectiveness Data and Information Set recommendations, with an improvement in the sexual health of the entire population associated with the earlier identification of infections and a reduction in transmission probabilities. In light of the recently released STI surveillance data from the CDC,^1^ changes in process are needed, and the new POC assay described here can be an addition to our toolbox. Future research will need to evaluate the performance of this POC assay for use in rectal and oropharyngeal samples to support CDC guidelines for screening among people taking HIV preexposure prophylaxis and men who have sex with men.

## Notes

Supplement. **eTable 1.** Concordance of Chlamydia Assay Results **eTable 2.** Comparison of Chlamydia Assay Performances **eTable 3.** Concordance of Gonorrhea Assay Results **eTable 4.** Comparison of Gonorrhea Assay Performance
