# Supplementary material for: Evaluation of the Performance of a Point-of-Care Test for Chlamydia and Gonorrhea
Source: JAMA Netw Open. 2020 May 14;3(5):e204819. doi: 10.1001/jamanetworkopen.2020.4819 (PMC7225902; doi:10.1001/jamanetworkopen.2020.4819)
Supplement: Supplement. — eTable 1. Concordance of Chlamydia Assay Results eTable 2. Comparison of Chlamydia Assay Performances eTable 3. Concordance of Gonorrhea Assay Results eTable 4. Comparison of Gonorrhea Assay Performance [file jamanetwopen-3-e204819-s001.pdf]

Supplementary Online Content

Van Der Pol B, Taylor SN, Mena L, et al. Evaluation of the performance of a point-of-care test for chlamydia and gonorrhea. *JAMA Netw Open.* 2020;3(5):e204819. doi:10.1001/jamanetworkopen.2020.4819

eTable 1. Concordance of Chlamydia Assay Results
eTable 2. Comparison of Chlamydia Assay Performances
eTable 3. Concordance of Gonorrhea Assay Results
eTable 4. Comparison of Gonorrhea Assay Performance

This supplementary material has been provided by the authors to give readers additional information about their work.

© 2020 Van Der Pol B, et al. *JAMA Network Open.*

eTable 1. Concordance of Chlamydia Assay Results

Chlamydia CIS	NAAT 1	NAAT 2	NAAT 3	binx *io*®	Symptom Status		Total
	Male Urine	Male Urine	Male Urine	Male Urine	Sx	Asx	
NI	-	-	-	-	243	542	785
NI	-	-	+	-	0	3	3
NI	-	+	-	-	0	0	0
NI	+	-	-	-	0	1	1
NI	-	-	IND	-	2	1	3
NI	-	IND	-	-	1	0	1
NI	IND	-	-	-	1	2	3
NI	-	-	-	+	1	5	6
NI	-	-	+	+	0	0	0
NI	-	+	-	+	0	0	0
NI	+	-	-	+	0	0	0
NI	-	-	IND	+	0	0	0
NI	-	IND	-	+	0	0	0
NI	IND	-	-	+	0	0	0
NI	-	-	-	IND	0	0	0
NI	-	-	+	IND	0	0	0
NI	-	+	-	IND	0	0	0
NI	+	-	-	IND	0	0	0
NI	-	-	IND	IND	0	0	0
NI	-	IND	-	IND	0	0	0
NI	IND	-	-	IND	0	0	0
**Total Not Infected**					248	554	802
I	+	+	+	+	54	56	110
I	+	+	-	+	0	0	0
I	+	-	+	+	0	0	0
I	-	+	+	+	0	0	0
I	+	+	IND	+	0	0	0
I	+	IND	+	+	0	0	0
I	IND	+	+	+	1	0	1
I	+	+	+	-	3	3	6
I	+	+	-	-	1	0	1
I	+	-	+	-	1	0	1
I	-	+	+	-	0	1	1
I	+	+	IND	-	0	0	0
I	+	IND	+	-	0	0	0
I	IND	+	+	-	0	0	0
I	+	+	+	IND	0	0	0
I	+	+	-	IND	0	0	0
I	+	-	+	IND	0	0	0
I	-	+	+	IND	0	0	0
I	+	+	IND	IND	0	0	0
I	+	IND	+	IND	0	0	0
I	IND	+	+	IND	0	0	0
**Total Infected**					60	60	120

Chlamydia CIS	NAAT 1	NAAT 2	NAAT 3	binx *io*®		Symptom Status		Total
	CCVS	CCVS	CCVS	collection	result	Sx	Asx	
NI	-	-	-	SCVS	-	350	308	658
NI	-	-	+	SCVS	-	1	1	2
NI	-	+	-	SCVS	-	5	3	8
NI	+	-	-	SCVS	-	0	0	0
NI	-	-	IND	SCVS	-	2	1	3
NI	-	IND	-	SCVS	-	0	1	1
NI	IND	-	-	SCVS	-	4	0	4
NI	-	-	-	SCVS	+	4	2	6
NI	-	-	+	SCVS	+	0	0	0
NI	-	+	-	SCVS	+	0	0	0
NI	+	-	-	SCVS	+	0	0	0
NI	-	-	IND	SCVS	+	0	0	0

© 2020 Van Der Pol B, et al. *JAMA Network Open.*

**NI**	-	IND	-	SCVS	+	0	0	0
**NI**	IND	-	-	SCVS	+	0	0	0
**NI**	-	-	-	SCVS	IND	0	0	0
**NI**	-	-	+	SCVS	IND	0	0	0
**NI**	-	+	-	SCVS	IND	0	0	0
**NI**	+	-	-	SCVS	IND	0	0	0
**NI**	-	-	IND	SCVS	IND	0	0	0
**NI**	-	IND	-	SCVS	IND	0	0	0
**NI**	IND	-	-	SCVS	IND	0	0	0
**NI**	-	-	-	CCVS	-	381	312	693
**NI**	-	-	+	CCVS	-	1	2	3
**NI**	-	+	-	CCVS	-	0	4	4
**NI**	+	-	-	CCVS	-	0	0	0
**NI**	-	-	IND	CCVS	-	0	0	0
**NI**	-	IND	-	CCVS	-	1	1	2
**NI**	IND	-	-	CCVS	-	2	1	3
**NI**	-	-	-	CCVS	+	4	3	7
**NI**	-	-	+	CCVS	+	0	0	0
**NI**	-	+	-	CCVS	+	0	0	0
**NI**	+	-	-	CCVS	+	0	0	0
**NI**	-	-	IND	CCVS	+	0	0	0
**NI**	-	IND	-	CCVS	+	0	0	0
**NI**	IND	-	-	CCVS	+	0	0	0
**NI**	-	-	-	CCVS	IND	0	0	0
**NI**	-	-	+	CCVS	IND	0	0	0
**NI**	-	+	-	CCVS	IND	0	0	0
**NI**	+	-	-	CCVS	IND	0	0	0
**NI**	-	-	IND	CCVS	IND	0	0	0
**NI**	-	IND	-	CCVS	IND	0	0	0
**NI**	IND	-	-	CCVS	IND	0	0	0
**Total Not Infected**						755	639	1394
**I**	+	+	+	SCVS	+	17	32	49
**I**	+	+	-	SCVS	+	0	1	1
**I**	+	-	+	SCVS	+	0	0	0
**I**	-	+	+	SCVS	+	1	0	1
**I**	+	+	IND	SCVS	+	0	0	0
**I**	+	IND	+	SCVS	+	1	0	1
**I**	IND	+	+	SCVS	+	0	0	0
**I**	+	+	+	SCVS	-	1	0	1
**I**	+	+	-	SCVS	-	1	0	1
**I**	+	-	+	SCVS	-	0	0	0
**I**	-	+	+	SCVS	-	0	0	0
**I**	+	+	IND	SCVS	-	0	0	0
**I**	+	IND	+	SCVS	-	0	0	0
**I**	IND	+	+	SCVS	-	0	0	0
**I**	+	+	+	SCVS	IND	0	0	0
**I**	+	+	-	SCVS	IND	0	0	0
**I**	+	-	+	SCVS	IND	0	0	0
**I**	-	+	+	SCVS	IND	0	0	0
**I**	+	+	IND	SCVS	IND	0	0	0
**I**	+	IND	+	SCVS	IND	0	0	0
**I**	IND	+	+	SCVS	IND	0	0	0
**I**	+	+	+	CCVS	+	39	31	70
**I**	+	+	-	CCVS	+	0	0	0
**I**	+	-	+	CCVS	+	0	1	1
**I**	-	+	+	CCVS	+	0	0	0
**I**	+	+	IND	CCVS	+	0	0	0
**I**	+	IND	+	CCVS	+	0	0	0
**I**	IND	+	+	CCVS	+	1	0	1
**I**	+	+	+	CCVS	-	0	2	2
**I**	+	+	-	CCVS	-	1	0	1
**I**	+	-	+	CCVS	-	0	0	0
**I**	-	+	+	CCVS	-	0	0	0
**I**	+	+	IND	CCVS	-	0	0	0
**I**	+	IND	+	CCVS	-	0	0	0

© 2020 Van Der Pol B, et al. *JAMA Network Open.*

**I**	IND	+	+	CCVS	-	0	0	0
**I**	+	+	+	CCVS	IND	1	0	1
**I**	+	+	-	CCVS	IND	0	0	0
**I**	+	-	+	CCVS	IND	0	0	0
**I**	-	+	+	CCVS	IND	0	0	0
**I**	-	-	IND	CCVS	IND	0	0	0
**I**	-	IND	-	CCVS	IND	0	0	0
**I**	IND	-	-	CCVS	IND	0	0	0
**Total Infected**						63	67	130

NI= Not Infected   I = Infected   IND- indeterminate

SCVS = self-collected vaginal swab   CCVS = Clinician collected vaginal swab

© 2020 Van Der Pol B, et al. *JAMA Network Open.*

eTable 2. Comparison of Chlamydia Assay Performances

Men

	binx	NAAT 1	NAAT 2	NAAT 3
**TP**	111	116	117	117
**FN**	9	1	1	1
**Sensitivity**	92.5%	99.1%	99.2%	99.2%
**95% Conf Int**	86.4% - 96.0%	95.3% - 99.8%	95.4% - 99.9%	95.4% - 99.9%
**TN**	796	798	801	796
**FP**	6	3	2	5
**Specificity**	99.3%	99.6%	99.8%	99.4%
**95% Conf Int**	98.4% - 99.7%	98.9% - 99.9%	99.1% - 99.9%	98.5% - 99.7%

Women

	binx	NAAT 1	NAAT 2	NAAT 3
**TP**	124	126	126	127
**FN**	5	1	1	3
**Sensitivity**	96.1%	99.2%	99.2%	97.7%
**95% Conf Int**	91.2% - 98.3%	95.7% - 99.9%	95.7% - 99.9%	93.4% - 99.2%
**TN**	1381	1387	1379	1386
**FP**	13	2	14	5
**Specificity**	99.1%	99.9%	99.0%	99.6%
**95% Conf Int**	98.4% - 99.5%	99.5% - 100.0%	98.3% - 99.4%	99.2% - 99.8%

© 2020 Van Der Pol B, et al. *JAMA Network Open.*

eTable 3. Concordance of Gonorrhea Assay Results

Gonorrhea CIS	NAAT 1	NAAT 2	NAAT 3	binx *io*®	Symptom Status		Total
	Male Urine	Male Urine	Male Urine	Male Urine	Sx	Asx	
NI	-	-	-	-	241	594	835
NI	-	-	+	-	1	4	5
NI	-	+	-	-	0	0	0
NI	+	-	-	-	1	2	3
NI	-	-	IND	-	2	1	3
NI	-	IND	-	-	1	0	1
NI	IND	-	-	-	0	1	1
NI	-	-	-	+	0	0	0
NI	-	-	+	+	0	0	0
NI	-	+	-	+	0	0	0
NI	+	-	-	+	0	0	0
NI	-	-	IND	+	0	0	0
NI	-	IND	-	+	0	0	0
NI	IND	-	-	+	0	0	0
NI	-	-	-	IND	0	0	0
NI	-	-	+	IND	0	0	0
NI	-	+	-	IND	0	0	0
NI	+	-	-	IND	0	0	0
NI	-	-	IND	IND	0	0	0
NI	-	IND	-	IND	0	0	0
NI	IND	-	-	IND	0	0	0
**Total Not Infected**					246	602	848
I	+	+	+	+	59	10	69
I	+	+	-	+	0	0	0
I	+	-	+	+	0	0	0
I	-	+	+	+	0	0	0
I	+	+	IND	+	0	0	0
I	+	IND	+	+	0	0	0
I	IND	+	+	+	2	1	3
I	+	+	+	-	0	1	1
I	+	+	-	-	0	0	0
I	+	-	+	-	1	0	1
I	-	+	+	-	0	0	0
I	+	+	IND	-	0	0	0
I	+	IND	+	-	0	0	0
I	IND	+	+	-	0	0	0
I	+	+	+	IND	0	0	0
I	+	+	-	IND	0	0	0
I	+	-	+	IND	0	0	0
I	-	+	+	IND	0	0	0
I	+	+	IND	IND	0	0	0
I	+	IND	+	IND	0	0	0
I	IND	+	+	IND	0	0	0
**Total Infected**					62	12	74

Gonorrhea CIS	NAAT 1	NAAT 2	NAAT 3	binx *io*®		Symptom Status		Total
	CCVS	CCVS	CCVS	Collection method	result	Sx	Asx	
NI	-	-	-	SCVS	-	371	334	705
NI	-	-	+	SCVS	-	0	1	1
NI	-	+	-	SCVS	-	0	0	0
NI	+	-	-	SCVS	-	0	0	0
NI	-	-	IND	SCVS	-	2	1	3
NI	-	IND	-	SCVS	-	0	1	1
NI	IND	-	-	SCVS	-	4	0	4
NI	-	-	-	SCVS	+	0	0	0
NI	-	-	+	SCVS	+	0	0	0
NI	-	+	-	SCVS	+	0	0	0
NI	+	-	-	SCVS	+	0	0	0

© 2020 Van Der Pol B, et al. *JAMA Network Open.*

**NI**	-	-	IND	SCVS	+	0	0	0
**NI**	-	IND	-	SCVS	+	0	1	1
**NI**	IND	-	-	SCVS	+	0	0	0
**NI**	-	-	-	SCVS	IND	0	0	0
**NI**	-	-	+	SCVS	IND	0	0	0
**NI**	-	+	-	SCVS	IND	0	0	0
**NI**	+	-	-	SCVS	IND	0	0	0
**NI**	-	-	IND	SCVS	IND	0	0	0
**NI**	-	IND	-	SCVS	IND	0	0	0
**NI**	IND	-	-	SCVS	IND	0	0	0
**NI**	-	-	-	CCVS	-	405	347	752
**NI**	-	-	+	CCVS	-	3	3	6
**NI**	-	+	-	CCVS	-	0	0	0
**NI**	+	-	-	CCVS	-	0	0	0
**NI**	-	-	IND	CCVS	-	0	0	0
**NI**	-	IND	-	CCVS	-	0	1	1
**NI**	IND	-	-	CCVS	-	2	1	3
**NI**	-	-	-	CCVS	+	0	0	0
**NI**	-	-	+	CCVS	+	0	0	0
**NI**	-	+	-	CCVS	+	0	0	0
**NI**	+	-	-	CCVS	+	1	0	1
**NI**	-	-	IND	CCVS	+	0	0	0
**NI**	-	IND	-	CCVS	+	0	0	0
**NI**	IND	-	-	CCVS	+	0	0	0
**NI**	-	-	-	CCVS	IND	1	0	1
**NI**	-	-	+	CCVS	IND	0	0	0
**NI**	-	+	-	CCVS	IND	0	0	0
**NI**	+	-	-	CCVS	IND	0	0	0
**NI**	-	-	IND	CCVS	IND	0	0	0
**NI**	-	IND	-	CCVS	IND	0	0	0
**NI**	IND	-	-	CCVS	IND	0	0	0
**Total Not Infected**						789	690	1479
**I**	+	+	+	SCVS	+	8	10	18
**I**	+	+	-	SCVS	+	1	0	1
**I**	+	-	+	SCVS	+	0	1	1
**I**	-	+	+	SCVS	+	1	0	1
**I**	+	+	IND	SCVS	+	0	0	0
**I**	+	IND	+	SCVS	+	0	0	0
**I**	IND	+	+	SCVS	+	0	0	0
**I**	+	+	+	SCVS	-	0	0	0
**I**	+	+	-	SCVS	-	0	0	0
**I**	+	-	+	SCVS	-	0	0	0
**I**	-	+	+	SCVS	-	0	0	0
**I**	+	+	IND	SCVS	-	0	0	0
**I**	+	IND	+	SCVS	-	0	0	0
**I**	IND	+	+	SCVS	-	0	0	0
**I**	+	+	+	SCVS	IND	0	0	0
**I**	+	+	-	SCVS	IND	0	0	0
**I**	+	-	+	SCVS	IND	0	0	0
**I**	-	+	+	SCVS	IND	0	0	0
**I**	+	+	IND	SCVS	IND	0	0	0
**I**	+	IND	+	SCVS	IND	0	0	0
**I**	IND	+	+	SCVS	IND	0	0	0
**I**	+	+	+	CCVS	+	16	5	21
**I**	+	+	-	CCVS	+	0	0	0
**I**	+	-	+	CCVS	+	2	0	2
**I**	-	+	+	CCVS	+	0	0	0
**I**	+	+	IND	CCVS	+	0	0	0
**I**	+	IND	+	CCVS	+	0	0	0
**I**	IND	+	+	CCVS	+	1	0	1
**I**	+	+	+	CCVS	-	0	0	0
**I**	+	+	-	CCVS	-	0	0	0
**I**	+	-	+	CCVS	-	0	0	0
**I**	-	+	+	CCVS	-	0	0	0
**I**	+	+	IND	CCVS	-	0	0	0

© 2020 Van Der Pol B, et al. *JAMA Network Open.*

**I**	+	IND	+	CCVS	-	0	0	0
**I**	IND	+	+	CCVS	-	0	0	0
**I**	+	+	+	CCVS	IND	0	0	0
**I**	+	+	-	CCVS	IND	0	0	0
**I**	+	-	+	CCVS	IND	0	0	0
**I**	-	+	+	CCVS	IND	0	0	0
**I**	-	-	IND	CCVS	IND	0	0	0
**I**	-	IND	-	CCVS	IND	0	0	0
**I**	IND	-	-	CCVS	IND	0	0	0
**Total Infected**						29	16	45

NI= Not Infected   I = Infected   IND- indeterminate
SCVS = self-collected vaginal swab   CCVS = Clinician collected vaginal swab
Sx = Symptomatic   Asx = Asymptomatic

© 2020 Van Der Pol B, et al. *JAMA Network Open.*

eTable 4. Comparison of Gonorrhea Assay Performance

Men

	**binx**	**NAAT 1**	**NAAT 2**	**NAAT 3**
**TP**	72	70	73	73
**FN**	2	0	1	0
**Sensitivity**	97.3%	100.0%	98.6%	100.0%
**95% Conf Int**	90.7% - 99.3%	94.8% - 100.0%	92.7% - 99.8%	95.0% - 100.0%
**TN**	848	844	847	840
**FP**	0	4	0	6
**Specificity**	100.0%	99.5%	100.0%	99.3%
**95% Conf Int**	99.5% - 100.0%	98.8% - 99.8%	99.5% - 100.0%	98.5% - 99.7%

Women

	**binx**	**NAAT 1**	**NAAT 2**	**NAAT 3**
**TP**	45	43	42	44
**FN**	0	1	4	2
**Sensitivity**	100.0%	97.7%	91.3%	95.7%
**95% Conf Int**	92.1% - 100.0%	88.2% - 99.6%	79.7% - 96.6%	85.5% - 98.8%
**TN**	1476	1470	1475	1467
**FP**	2	1	0	7
**Specificity**	99.9%	99.9%	100.0%	99.5%
**95% Conf Int**	99.5% - 100.0%	99.6% - 100.0%	99.7% - 100.0%	99.0% - 99.8%

© 2020 Van Der Pol B, et al. *JAMA Network Open.*
